# Impact of Metabolic Syndrome and Its Components on Clinical Severity and Long-Term Prognosis in Patients With Premature Myocardial Infarction

**DOI:** 10.3389/fendo.2022.920470

**Published:** 2022-06-30

**Authors:** Jing Gao, Yuan Wang, Ya-Nan Yang, Xiao-Yuan Wu, Yan Cui, Zhong-He Zou, Zhuang Cui, Yin Liu

**Affiliations:** ^1^ Chest Hospital, Tianjin University, Tianjin, China; ^2^ Thoracic Clinical College, Tianjin Medical University, Tianjin, China; ^3^ Cardiovascular Institute, Tianjin Chest Hospital, Tianjin, China; ^4^ Cancer Department, Daping Hospital, Army Medical University, Chongqing, China; ^5^ Epidemiology and Biostatistics Institute, School of Public Health, Tianjin Medical University, Tianjin, China; ^6^ Department of Cardiology, Tianjin Chest Hospital, Tianjin, China

**Keywords:** metabolic syndrome, premature myocardial infarction, clinical severity, long-term prognosis, MACE

## Abstract

**Background:**

The effects of metabolic syndrome (MS) on premature myocardial infarction (PMI) are not clear to date. This study aimed to investigate the impact of MS and its components on clinical severity and long-term prognosis in patients with PMI.

**Methods:**

We enrolled 772 patients aged ≤45 years old who were diagnosed with acute myocardial infarction (AMI) at our hospital consecutively between 2015 and 2020. The patients were divided into an MS group and non-MS group. The parameters of clinical severity were compared using regression analysis. Patients were followed for median of 42 months for major adverse cardiovascular events (MACE).

**Results:**

Hyperglycemia was associated with multi-vessel disease [odds ratio*(OR)*=1.700, *95%* confidence interval (*CI)*=1.172-2.464, *P*=0.005] and Syntax score ≥33 (*OR*=2.736, *95% CI*=1.241-6.032, *P*=0.013). Increased MACE were observed in the MS group(17.9% vs 10.3%, *P*=0.004).The Kaplan-Meier curve also showed significant differences (*P*< 0.001). MS was an independent risk factor for MACE. Of each component of MS, BMI ≥28 kg/m^2^ (hazard ratio [*HR*]=2.022, *95% CI* =1.213-3.369, *P*=0.007] and hyperglycemia (*HR=2.904, 95% CI=1.847-4.567, P<0.001*) were independent risk factors for MACE.

**Conclusions:**

In patients with PMI, 1) hyperglycemia usually indicates more severe lesions; 2) MS as a whole was an independent risk factor for MACE; 3) BMI ≥28.0 kg/m^2^ and hyperglycemia were associated with MACE.

## Introduction

Metabolic syndrome (MS) is classified as a collection of risk factors, including hyperglycemia, atherogenic dyslipidemia, central obesity, hypertension, prothrombotic and proinflammatory states, that increase the risk of coronary artery disease (CAD), cardiovascular disease (CVD) and diabetes mellitus type 2 (1). It is estimated that MS affects about 1/4 of the global population and has become a major threat to public health in contemporary society (2).

With the rapid development of the social economy, the lifestyle of young people has also undergone great changes, including that lack of exercise, irregular eating habits. And also, often staying up late has become a common phenomenon among today’s young people. As a result, the population of obesity and hyperglycemia in China is increasing year by year, while both of them are important components of MS. Therefore, the individuals with MS are more likely to be younger (3). MS is highly prevalent in younger adults (<45 years) with acute myocardial infarction(AMI) (4–6). At the same time, the prevalence and mortality of myocardial infarction also follows a younger trend (7–10).This indicates that MS may play an important role in the development of AMI in this population. At present, no unified standard has been established on the age limit of premature myocardial infarction(PMI) at home and abroad, but most of the existing studies on AMI in younger adults have set the age at 45 years (11–13), so this study includes the adult population below the cut-off point of 45 years.

PMI leads to premature incapacity to work, increases public health care costs, and creates a huge social and economic burden. Studies at home and abroad have shown that MS increases the risk of coronary heart disease and death, and is associated with a poor prognosis and occurrence of MACE in patients aged >45 years with AMI (3, 14–17). Moreover, few studies have focused on the impact of MS on the severity of AMI lesions in patients >45 years of age, and the results are also different (14, 18). However, there is a lack of research on the effect of MS on PMI (≤45 years). Therefore, the purpose of this study was to investigate the impact of MS and its components on the severity of coronary artery lesions and long-term prognosis in patients with PMI.

## Methods

### Study Design and Participants

Consecutive patients ≤45 years of age who were diagnosed with AMI in Tianjin Chest Hospital from 2015 to 2020 were included. We diagnosed AMI according to the fourth edition of the global definition of myocardial infarction (19): Clinical evidence of acute myocardial injury accompanied by acute myocardial ischemia, that is, cTn is detected to be worthy of increase and or decrease (at least once exceeding the upper limit of 99 percentile),and at least one of the following: 1) symptoms of myocardial ischemia; 2) new ischemic ECG changes; 3) pathological Q waves; 4) imaging evidence that new patterns of myocardial inactivation or new focal wall motion abnormalities are consistent with ischemic pathological changes; and 5) coronary thrombosis found by radiography or autopsy. After excluding patients with severe liver or kidney diseases, malignant tumors and lack of diagnostic data related to MS, 772 patients were finally included.

### Ethical Considerations

The study protocol was approved by the Internal Review Board of Tianjin Chest Hospital (No. 2017KY-007-01) and all included patients provided signed informed consent prior to study participation. All procedures performed were in accordance with the ethical standards of the Helsinki Declaration and its later amendments, or comparable ethical standards.

### Diagnostic Criteria of MS

MS was defined according to the criteria of the 2005 National Cholesterol Education Program (20). In detail, the definition of MS requires the existence of any 3 of the following 5 criteria: 1) Hypertension: blood pressure ≥130/85 mmHg or consistent hypertensive patients undergoing treatment; 2) Hypertriglyceridemia: fasting plasma triglyceride ≥1.7 mmol/L; 3) Fasting HDL -cholesterol <1.0 mmol/L in men and <1.3 mmol/L in women. 4) Hyperglycemia: fasting blood glucose level ≥6.1 mmol/L or known diabetic patients undergoing treatment; 5) Central obesity: waist circumference >90 cm for men and >80 cm for women. Since the waist circumference of the patient was not measured in this study, we used BMI ≥28.0 kg/m^2^ as the diagnostic criterion of obesity as proposed by the working group on obesity in China (WGOC) (21).

### Clinical and Biochemical Measurements

The basic data of sex, age, BMI and medical history of all patients were recorded on admission, and the admission conditions of the patients were evaluated, including sitting blood pressure (measured by senior doctors on the non-dominant arm supported by the heart level), Killip class and the type of AMI. The emergency laboratory indexes of admission (hypersensitive troponin T, CK, CK-MB, etc.) were recorded. After fasting overnight for 12 hours, fasting blood glucose (FBG), triglyceride (TG), high density lipoprotein cholesterol (HDL-C), low density lipoprotein cholesterol (LDL-C) and total cholesterol were measured. Coronary angiography and PCI were performed by an independent physician with qualification for coronary artery diagnosis and treatment in Tianjin Chest Hospital and multi-position projection head position was used in coronary angiography. Postoperative antiplatelet therapy with aspirin 100 mg/d and clopidogrel 75 mg/d or ticagrelor 90 mg twice per day was recommended for at least one year. The Syntax score, which is widely used to evaluate the severity of coronary artery disease, as well as to guide risk stratification and revascularization strategies in patients with coronary heart disease (22), was calculated for the present study by two or more physicians with experience performing percutaneous coronary intervention (PCI) who were blinded to the study and the patient’s condition. For risk stratification of the severity of the disease based on Syntax scores, patients with scores ≤ 22 were at low risk, scores 23-32 were at moderate risk, and those ≥ 33 were at high risk. According to clinical standards and current echocardiographic guidelines, all patients had at least one echocardiographic examination in the first week after AMI (23).

### Follow-Up and Outcomes

The patients were followed after discharge by telephone and/or by interview after the initial appointment by trained nurses or cardiologists blinded to laboratory test results, The average follow-up time was 42 months. The end points were defined as MACE, which was a composite outcome consisting target vessel revascularization (TVR), re-hospitalization for heart failure (HF), cardiac death, recurrent myocardial infarction and stroke. TVR is defined as ischemic symptoms or event-driven revascularization of any lesion, including PCI and CABG, which is unplanned. Heart failure was diagnosed according to the guidelines from the European Society of Cardiology (24). Cardiac death is mainly caused by sudden cardiac death, congestive heart failure, AMI, severe arrhythmia, stroke or other structural/functional heart diseases. Myocardial infarction was diagnosed comprehensively by symptoms such as chest pain, changes in myocardial enzymes and electrocardiogram results. Stroke was defined as acute cerebral infarction according to imaging results or typical symptoms. All outcomes were adjudicated centrally by two independent cardiologists, and any disagreement was resolved by consensus.

### Statistical Analysis

Continuous variables were tested for normality using the Kolmogorov-Smirnov test. The continuous data of normal distribution were expressed as mean ± standard deviation, the comparison between the two groups was performed by independent student t test, the continuous data of skewness distribution was expressed by M (Q1 ~ Q3), and the comparison between the two groups was performed by Mann Whitney U test. The categorical data were expressed as frequency and percentage, and the comparison between the two groups was made by Chi-square test or Fisher exact probability method (when the theoretical frequency< 1 or the number of cases <40). Multivariate logistic regression model was used to analyze the effects of MS and its components on lesion severity. The survival curves of MS patients and non-MS patients were drawn using the Kaplan-Meier method and were compared with the logarithmic rank test. Cox proportional hazards models were adjusted for age, sex, smoking history, family history of coronary heart disease, Killip ≥II, left ventricular ejection fraction (LVEF)< 40%, Syntax score ≥33, multi-vessel disease, PCI, the use of ACEI/ARB and Beta-blocker. The hazard ratios (HR) of MS and its five components were calculated to evaluate their effects on MACE. To evaluate the incremental prognostic value of MS and two of its components (BMI ≥28.0 kg/m^2^, hyperglycemia) on MACE, we established model 1 (basic model) to include the above 11 variables. Model 2a includes basic model + BMI ≥28.0 kg/m^2^. Model 2b includes variables in the basic model plus hyperglycemia. Model 3 includes the basic model + MS, we compared the AUC difference, calculate NRI,(Net Reclassification) and IDI(Integrated Discrimination Improvement) between ① model 3 and model 2a and ② model 3 and model 2b. Bilateral *P <*0.05 was established as statistical significance. All statistical analyses were determined using SPSS software version 26.0 (IBM SPSS Statistics, Chicago, IL, USA) and R software (version 4.2.0).

## Results

### Comparison of Baseline Characteristics

After excluding patients with severe liver or kidney diseases, malignant tumors and lack of diagnostic data related to MS, 772 patients were finally included. As shown in **Table 1**, among the 772 patients included in this study, 417 (54%) met the diagnostic criteria of MS and 355 (46%) were in the non-MS group. Males accounted for the majority of the two groups. Compared with the non-MS group, the MS group had more history of diabetes, hypertension, smoking and chronic kidney disease. In terms of MS and its components, the BMI value of the MS group was higher than that of the non-MS group, and the proportion of BMI ≥ 28.0 kg/m^2^, hypertension, hyperglycemia, hypertriglyceridemia and low HDL-C was significantly higher than that of the non-MS group (*P*< 0.001) (**Supplementary Figure** **)**. In the MS group, the most common MS component was hypertension (90.2%), followed by low HDL-C (89%), hypertriglyceridemia (88.2%), hyperglycemia (65.2%) and BMI ≥28.0 kg/m^2^ (24.5%) (**Supplementary Figure**). On admission, systolic and diastolic blood pressure values were higher in the MS group than in the non-MS group. For type of MI, NSTEMI was the most common type in the MS group (32.1% vs 22.5%, *P*=0.003), while STEMI was more frequent in the non-MS group (77.5% vs 67.9%, *P*=0.003). Among laboratory indexes, the levels of fasting glucose, hypersensitive C-reactive protein, total cholesterol and triglyceride were higher in the MS group than those in the non-MS group, while the level of HDL-C in the MS group was significantly lower than that in non-MS group.

**Table 1:** Demographic and clinical characteristics of patients with premature myocardial infarction.

Variables	non-MS group (n = 355)	MS group (n = 417)	*P*
Age (year)	41 (36-43.3)	41 (38-43)	0.616
Male,n (%)	335 (94.4)	405 (97.1)	0.056
BMI (kg/m^2^)	24.8 (23.1-26.2)	25.9 (23.9-27.9)	P<0.001
**Medical history**			
History of Diabetes, n, (%)	24 (6.8)	138 (33.1)	*P*<0.001
History of Hypertension, n, (%)	112 (31.5)	254 (60.9)	*P*<0.001
Previous angina pectoris, n, (%)	75 (21.1)	97 (23.3)	0.477
History of MI, n, (%)	14 (3.9)	22 (5.3)	0.382
Family history of CVD,n, (%)	57 (16.1)	60 (14.4)	0.52
History of smoking,n, (%)	265 (74.4)	339 (81.3)	0.026
History of drinking,n, (%)	155 (43.7)	167 (40)	0.31
Renal insufficiency,n, (%)	1 (0.3)	9 (2.2)	0.022
Previous PCI,n, (%)	11 (3.1)	12 (2.9)	0.857
Previous CABG,n, (%)	1 (0.1)	2 (0.3)	1
Cerebrovascular disease,n, (%)	11 (3.1)	13 (3.1)	0.988
**Admission**			
Systolic blood pressure (mmHg)	120 (110-145)	133 (125-151.5)	*P*<0.001
Diastolic blood pressure (mmHg)	75 (69.8-87.8)	85.5 (70-94.3)	*P*<0.001
Killip class			0.627
I	343 (96.6)	407 (97.6)	
II	9 (2.5)	6 (1.4)	
III	2 (0.6)	3 (0.7)	
IV	1 (0.3)	1 (0.2)	
Killip ≥II, n, (%)	12 (3.4)	10 (2.4)	0.414
**Type of MI**			
STEMI, n, (%)	275 (77.5)	283 (67.9)	0.003
NSTEMI, n, (%)	80 (22.5)	134 (32.1)	0.003
**Laboratory**			
LVEF<40%, n, (%)	22 (6.2)	22 (5.3)	0.594
LVEF (%)	53 (48-58)	53 (47-58)	0.47
Fasting blood glucose (mmol/L)	5.1 (4.6-5.6)	6.7 (5.3-8.4)	*P*<0.001
CK (U/L)	1728 (725.3-3064.8)	1599.5 (630.3-3020)	0.126
CK MB (U/L)	159.5 (47.3-258.3)	122 (52.8-229.3)	0.111
Hypersensitive C-reactive protein (mmol/L)	5.3 (2.4-15)	6.3 (3.2-14.6)	0.045
Total cholesterol (mmol/L)	4.8 ± 1.2	5.0 ± 1.2	0.002
Triglyceride (mmol/L)	1.5 (1.2-2.1)	2.5 (2-3.5)	*P*<0.001
HDL-C (mmol/L)	1 (0.9-1.2)	0.9 (0.7-0.9)	*P*<0.001
Hypersensitive troponin T (ug/L)	2.9 (1-5.7)	3.2 (1.1-6.8)	0.206
LDL-C (mmol/L)	3.1 (2.5-3.8)	3.2 (2.6-3.9)	0.215
**CAG and treatment**			
CAG, n, (%)	332 (93.5)	394 (94.5)	0.573
Conservative treatment, n (%)	51 (14.4)	61 (14.6)	0.918
Thrombolysis, n (%)	6 (1.7)	5 (1.2)	0.566
CABG, n (%)	6 (1.7)	6 (1.4)	0.778
PCI, n (%)	292 (82.3)	345 (82.7)	0.861
**Severity of coronary artery lesion**, n (%)			
Single vessel disease	151 (45.5)	147 (37.3)	0.026
Two-vessel disease	90 (27.1)	115 (29.2)	0.535
Three-vessel disease	81 (24.4)	126 (32.0)	0.024
Left main	10 (3.0)	6 (1.5)	0.173
multi-vessel disease	181 (54.5)	247 (62.7)	0.026
Syntax score	16 (9-22)	15.5 (10-22.5)	0.526
Syntax ≤ 22	252 (75.9)	289 (73.4)	0.432
Syntax (23-32)	50 (15.1)	60 (15.2)	0.950
Syntax≥33	19 (5.7)	25 (6.3)	0.726
**Baseline medication, n, (%)**			
DAPT	353 (99.4)	410 (98.3)	0.150
Beta-blocker	281 (79.2)	332 (79.6)	0.874
ACEI/ARB	242 (68.2)	273 (65.5)	0.427
Statin	353 (99.4)	410 (98.3)	0.150
Anticoagulants	341 (96.1)	389 (93.3)	0.091

MS, metabolic syndrome; MI, myocardial infarction; CVD, cardiovascular disease; PCI, percutaneous coronary intervention; CABG, coronary artery bypass graft; STEMI, ST-segment elevation myocardial infarction; NSTEMI, non-ST segment elevation myocardial infarction; CK, creatine kinase; CK-MB, creatine kinase isoenzyme; HDL-C, high density lipoprotein cholesterol; LDL-C, low density lipoprotein cholesterol; CAG, coronary angiography; DAPT, dual antiplatelet; ACEI/ARB, angiotensin-converting enzyme inhibitor/angiotensin receptor blocker.

### Coronary Angiography and Treatment

Among the 772 patients, 726 (94%) underwent CAG. In terms of treatment, 112 (14.5%) patients received conservative drug therapy, 11 (1.4%) patients received thrombolytic therapy, and 12 (1.6%) patients underwent CABG; a total of 637 (82.5%) patients received percutaneous coronary intervention (PCI). There was no significant difference in the above treatments between MS group and non-MS group(*P*>0.05). Among the patients undergoing CAG, single-vessel disease was dominant in the non-MS group (45.5% vs 37.3%, *P*=0.026), and the proportion of three-vessel disease and multi-vessel disease in the MS group was significantly higher than that in the non-MS group (32.0% vs 24.4%, *P*=0.024; 62.7% vs 54.5%*, P*=0.026). No differences were noted in Syntax scores between the two groups (**Table 1**).

### Comparison of Logistic Regression Analysis of Clinical Severity Between the Two Groups

**Figure 1:**
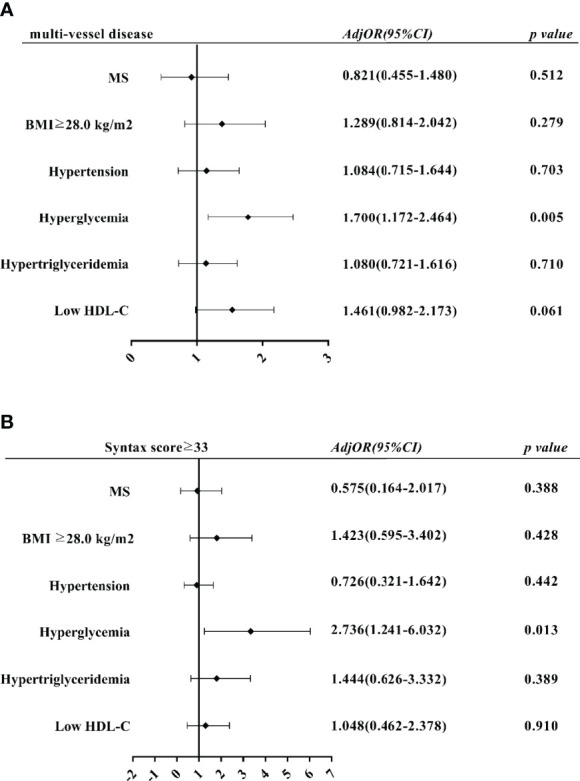
Multivariate logistic regression analysis of impact of MS components on PMI severity. **(A)** Effects of MS and its components on multi-vessel disease. **(B)** Effects of MS and its components on Syntax score ≥33 Adj OR, adjusted odds ratio; CI, confidence interval.

Multi-vessel disease and Syntax scores ≥33 were taken as dependent variables, MS and its five components were divided into independent variables for univariate and multivariate Logistic regression analysis (**Figure 1**). The results suggest that hyperglycemia is an independent risk factor for multi-vessel disease in the multivariate regression model (*OR*=1.700, *95% CI*=1.172-2.464, *P*=0.005) (**Figure 1A**). In the multivariate regression model with Syntax scores ≥33 as dependent variables, hyperglycemia was also an independent risk factor (*OR*=2.736, *95% CI*=1.241-6.032, *P*=0.013) (**Figure 1B**).

### Comparison of Clinical Prognosis Between the Two Groups

Patients were followed for median of 42 months for major adverse cardiovascular events (MACE), a total of 63(8.2%) people were lost to follow-up. We compared the baseline characteristics between patients lost to follow-up (n=63) and all (n=772) patients. The results exhibited that patients’ baseline characteristics were not significantly different between the two groups (all *P >*0.05, **Supplementary Table**). This shows that the people who lost follow-up have no effect on the conclusion of this study. After follow-up, 102 (14.4%) patients had MACE, including 68 (9.6%) cases of TVR, 20 (2.8%) cases of heart failure, 10 (1.4%) cases of cardiac death, 33 (4.7%) cases of recurrent MI, and 7 (1.0%) cases of stroke. A significant difference was found in the occurrence of MACE between the MS and non-MS groups (17.9% vs 10.3%, *P*=0.004), mainly due to significantly higher TVR and HF in the MS group than in the non-MS group (12.1% vs 6.7%, *P*=0.014; 4.7% vs 0.6%, *P*=0.001) (**Table 2**). In addition, AMI patients with prior PCI, prior CABG, LVEF <40%, high fasting blood glucose, high CK, high CK-MB, high hs-CRP, high hs-TNT, multi-vessel disease, or Syntax score ≥33 were positively associated with the occurrence of MACE (all *P*<0.05, **Supplementary Table**).

**Table 2:** Comparison of clinical outcomes between MS group and non-MS group.

Variables	non-MS group (n = 330)	MS group (n = 379)	*P*
MACE, n, (%)	34 (10.3)	68 (17.9)	0.004
TVR, n, (%)	22 (6.7)	46 (12.1)	0.014
HF, n, (%)	2 (0.6)	18 (4.7)	0.001
Cardiac death, n, (%)	4 (1.2)	6 (1.6)	0.676
Recurrent MI, n, (%)	14 (4.2)	19 (5.0)	0.627
Stroke, n, (%)	3 (0.9)	4 (1.1)	1.000

MS, metabolic syndrome; MACE, major adverse cardiovascular events; TVR, target vessel revascularization; HF, re-hospitalization for heart failure; MI, myocardial infarction.

**Figure 2:**
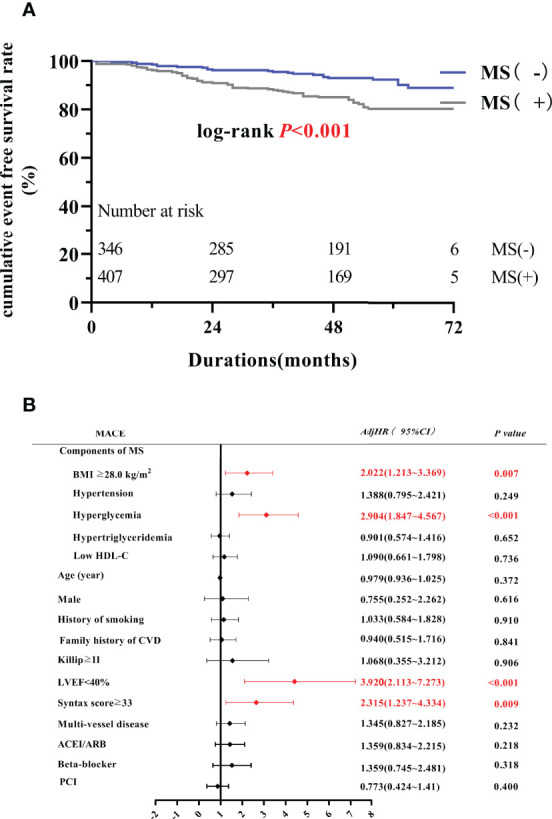
MS and PMI prognosis. **(A)** The Kaplan-Meier curve between MS group and non-MS group. **(B)** Multivariate Cox regression analysis of MACE. MACE, major adverse cardiovascular events; LVEF, left ventricular ejection fraction; ACEI/ARB, angiotensin converting enzyme inhibitor/angiotensin II receptor blocker; AdjHR, adjusted hazard ratio; CI, confidence interval; PCI, percutaneous coronary intervention.

A significant difference was found in the KM curve between the two groups (*P* < 0.001) (**Figure 2A**). Cox regression analysis showed that MS was an independent risk factor for MACE (*HR*=2.082, 95% *CI*=1.324-3.273, *P*=0.001), mainly due to the increased risk of TVR (*HR*=1.964, 95% *CI*=1.155-3.340, *P*=0.013) and HF (*HR*=8.301, *95% CI*=1.798 -38.332, *P*=0.007) (**Table 3**). Subsequent COX regression analysis of each component of MS showed that BMI ≥28.0 kg/m^2^ (*HR*=2.022, *95% CI*=1.213-3.369, *P* =0.007) and hyperglycemia (*HR*=2.904, *95% CI*=1.847-4.567, *P*<0.001) were independent risk factors for the occurrence of MACE (**Figure 2B**). In addition, LVEF <40% (*HR*=3.920, 95% *CI*=2.113-7.273, *P*<0.001) and Syntax scores ≥33(*HR*=2.315, 95% *CI*=1.237-4.334, *P*=0.009) were also independent risk factor for the occurrence of MACE.

**Table 3:** COX multivariate regression analysis of each adverse event in patients with PMI and MS.

Variables	*HR*	*95%CI*	*P*
MACE	2.082	1.324-3.273	0.001
TVR	1.964	1.155-3.340	0.013
HF	8.301	1.798-38.332	0.007
Cardiac death	2.355	0.351-15.801	0.378
Recurrent MI	1.360	0.645-2.867	0.419
Stroke	1.216	0.162-9.119	0.849

MS, metabolic syndrome; MACE, major adverse cardiovascular events; TVR, target vessel revascularization; HF, re-hospitalization for heart failure; MI, myocardial infarction HR, hazard ratio; CI, confidence interval.

**Figure 3:**
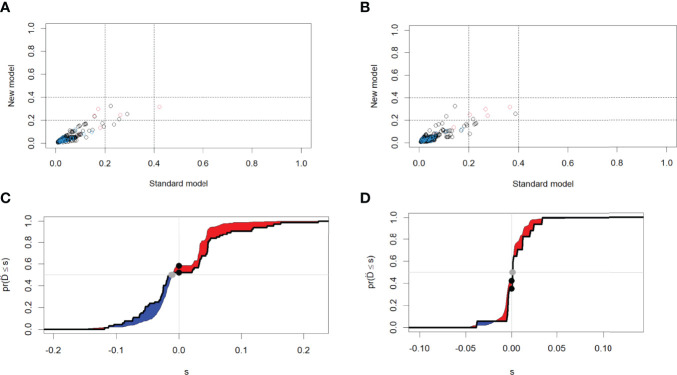
NRI and IDI between ① model 3 and model 2a ② model 3 and model 2b. **(A)**NRI for **① (B)** NRI for **② (C)** IDI for**① (D)** IDI for **②** NRI, Net Reclassification; IDI, Integrated Discrimination Improvement.

The analysis of the incremental prognostic value of MS and its components on MACE showed the AUC values of model 1, model 2a and 2b were 0.6381, 0.6751 and 0.6847, respectively, and the AUC of model 3 was 0.6665, which indicated that MS, BMI ≥28.0 kg/m^2^ and hyperglycemia all increased the predictive value of MACE. Furthermore, the NRI and IDI of AUC between model 3 and model 2a and between model 3 and model 2b were calculated. The NRI of model 3 vs model 2a and model 3 vs model 2b were 0.0411 and 0.0029 respectively, and the IDI of model 3 vs model 2a and model 3 vs model 2b was 0.008, 0.003, respectively. The results show that model 3 has better ability to predict MACE than model 2, that is, MS as a whole can better predict the poor prognosis of people with premature myocardial infarction (**Figure 3**).

## Discussion

Results of the present study highlights that: 1) In the PMI population of the present study, the prevalence rate of MS was 54%, and males accounted for the majority. NSTEMI was dominant in the MS group, while STEMI was higher in the non-MS group; 2) No correlations were found between MS as a whole and the severity of coronary artery lesions, but hyperglycemia in the MS components often predicted more severe coronary heart disease; 3) After median 42 months of follow-up, the incidence of MACE in the MS group was significantly higher than that found in the non-MS group, and MS was an independent risk factor for MACE, especially for the occurrence of TVR and HF. MS components, including BMI ≥28.0 kg/m^2^ and hyperglycemia, were independently associated with the occurrence of MACE in these patients.

MS is common in patients with coronary heart disease, especially in patients with AMI (15, 17, 25). Myocardial infarction is now showing a younger trend, affecting adults in middle age and younger (7–10). Studies have been conducted previously on the prevalence and distribution of MS components in people with PMI (4, 26, 27). A case-control study conducted by Kazemi et al. (26)in the AMI population ≤50 years old found that the prevalence of MS in the case group was significantly higher than that in the control group (34.7% vs 16.3%, respectively), and high triglycerides were the most common component. Gadepalli et al (4)estimated that the prevalence rate of MS in AMI patients ≤45 years old was about 62.74%, and the decrease in HDL level was the most common indicator. A meta-analysis of 34 large studies(age range 18–30 years old) suggested that MS was found in 4.8%-7% of younger adults (27). In that study, atherosclerotic dyslipidemia defined by low levels of HDL-C was the most common MS component (26.9%-41.2%), followed by elevated blood pressure (16.6%-26.6%), abdominal obesity (6.8%-23.6%), elevated triglycerides (8.6%-15.6%) and elevated fasting blood glucose (2.8%-15.4%). In the present study, MS accounted for 54% of people with PMI, and the most common component was hypertension (90.2%), followed by low HDL-C (89%), hypertriglyceridemia (88.2%), hyperglycemia (65.2%) and BMI ≥28.0 kg/m^2^ (24.5%). This results basically consistent with the results of previous studies, that is, MS accounts for about half of the patients with PMI, and dyslipidemia accounts for a large proportion.

MS has been reported to increase the risk of cardiovascular disease. Mottillo et al. (28) conducted a meta-analysis of 87 prospective studies, and the final results showed that MS was associated with an increased risk of cardiovascular disease with an *RR* of 2.35 (*95% CI*=2.02-2.73). Although the exact pathogenesis of MS is not completely clear, current studies suggest that MS increases the risk of cardiovascular disease in the following ways (29–32) (1): Insulin resistance: insulin resistance increases the entry of free fatty acids into the liver and increases the synthesis of triglyceride and very low-density lipoprotein. Compensatory hyperinsulinemia damages vascular endothelium and is conducive to lipid deposition, thus leading to the occurrence and development of atherosclerosis (2); Obesity and chronic inflammation: fat metabolites are released into the blood of obese patients, resulting in the accumulation of lipid metabolites, the penetration and activation of macrophages, and the chronic inflammatory state of the body, decreased adiponectin levels and increased interleukin-6 and plasminogen activator inhibitors, resulting in a state of high inflammation and high thrombosis.

MS not only increases the risk of CVD, especially AMI, but also affects the disease severity and prognosis of AMI patients (>45 years). These studies connect MS with more severe CAD. According to Mornar et al (33), more multi-vessel lesions are found in patients with MS. This observation was also noted in the present PMI population. In addition, Lovic et al. (14) used Syntax scores to assess the severity of coronary heart disease between patients with and without MS, and the results showed that patients with MS had higher Syntax scores. According to several studies, the higher the Syntax score, the worse the prognosis (34–36) (37). The present study expanded these findings and further demonstrated this observation in PMI patients, because in the long run (median 42 months of follow-up), Syntax scores ≥33 was independently associated with the occurrence of MACE. Although no differences were found in Syntax scores between groups, in order to evaluate the severity of lesions between the MS group and non-MS group, we classified the disease into low risk (≤22), moderate risk 23-32 and high risk (≥ 33) according to Syntax scores. As far as we know, this is the first study to apply this classification to delineate risk.

In the setting of ACS, the prognostic value of assessing the presence of MS as a comprehensive diagnosis remains uncertain (38–41). In fact, the various components of MS have different relationships with the outcome, which may affect the observed incremental risk stratification (38, 41, 42). In the present study, we found that MS as a whole can better predict the poor prognosis of people with premature myocardial infarction.

In present study, hyperglycemia in the MS components is an independent risk factor for multi-vessel disease and Syntax ≥33 in PMI patients Nevertheless, inconsistent with some previous studies, multivariate regression analysis adjusted by MS and five components did not find that dyslipidemia was associated with the severity of coronary artery disease. The possible reasons are as follows. Firstly, earlier studies have shown that diabetes is a challenging factor for the effects of MS on the severity of AMI, because it can independently increase the severity of coronary heart disease (43). The definition of hyperglycemia in MS diagnostic criteria also includes people with diabetes. So when MS and its components are included in multivariate adjustment analysis, hyperglycemia has become the main and robust factor affecting coronary artery disease. Secondly, it may be because we include people in the same area, and there is no significant difference in eating habits. However, it is worth noting that hyperglycemia in MS includes not only people with diabetes, but also patients with fasting blood glucose ≥6.1 mmol/L, some of whom may be in a prediabetic state or in a stage of insulin resistance. Levantesi et al. (44) pointed out that MS is a powerful predictor of delayed diabetes after myocardial infarction. Therefore, when clinicians diagnose MS as a whole, we pay particular attention to hyperglycemia, which in the present study usually indicated a more serious degree of coronary artery lesions; this is therefore helpful for identifying high-risk PMI patients with poor prognosis as early as possible, allowing implementation of more timely intervention measures to improve the prognosis of these younger adult patients. Therefore, we suggest that MS can serve as an early indicator of increased risk for PMI.

After median 42 months of follow-up, we found that MS was independently associated with MACE in PMI patients, which was consistent with the results of previous studies (>45 years) (3, 14, 45). We also found that this was due to the increased risk of TVR and HF, which is also consistent with previous studies of patients >45 years (25). In the present study, we found that among the MS components, hyperglycemia and BMI ≥28.0 kg/m^2^ were independent risk factors for MACE. Hyperglycemia induces the expression of cytokines and inflammatory factors, induces the production of growth factors that promote restenosis, smooth muscle cell proliferation and extracellular matrix production, and eventually leads to intimal hyperplasia and restenosis (46). Therefore, the revascularization rate in the MS group was significantly higher than that in the non-MS group. Also, previous studies have shown that MS is a major related factor in the risk of developing severe heart failure (47, 48), which is also observed in our population. In addition, obese patients are in a state of chronic inflammation and prethrombotic state, which plays an important role in the process of atherosclerosis and will lead to adverse cardiovascular events during follow-up (47). Early research has confirmed that abdominal obesity is independently associated with cardiovascular disease (49, 50). Consistent with our study results, Azarfarin et al. (51)pointed out that the left ventricular function was decreased in patients with abnormal BMI (*P*<0.05), suggesting that anthropometric indicators (e.g., BMI, waist circumference) have an impact on cardiac function after MI. Also, as mentioned above, the present study has shown that hyperglycemia in MS is independently associated with multi-vessel disease and Syntax score ≥33, while previous studies have shown that they all predict a poor prognosis (34, 52), which has been confirmed in long-term follow-up of the included PMI patients.

## Conclusions

Previous studies have confirmed that MS affects the occurrence, development and prognosis of acute myocardial infarction. As far as we know, this is the first observational study to explore the impact of MS on the clinical severity and prognosis of PMI in adults aged 45 years and younger, suggesting that MS component hyperglycemia is as an independent predictor for coronary artery lesions and MACE. This may be useful for improving the awareness of adults aged 45 years and younger about risk factors for PMI, MS and MACE, and to encourage starting intervention measures as early as possible to help prevent PMI and MS.

## Limitations

The present study has several limitations, including: 1) This was a single-center observational study and the sample size does not represent the overall prevalence of MS in patients with PMI; 2) The blood glucose and blood lipids measured during the onset of MI may be different from the daily levels, which may affect the diagnosis of MS; 3) The use of BMI instead of abdominal circumference to diagnose MS will likely have an impact on the results; 4) MS is independently related to the occurrence of MACE, but the results of exploratory analysis of each event of MACE, especially the occurrence of cardiac death, need to be further confirmed by larger sample size and longer follow-up time.

## Data Availability Statement

The original contributions presented in the study are included in the article/**Supplementary Material**. Further inquiries can be directed to the authors.

## Author Contributions

(I) Conception and design: JG YW and YL; (II) Administrative support: None; (III) Provision of study materials or patients: JG and YL; (IV) Collection and assembly of data: YW, X-YW, YC, Z-HZ and Y-NY; (V) Data analysis and interpretation: ZC, JG and YW; (VI) Manuscript writing: All authors; (VII) Final approval of manuscript: All authors.

## Funding

This research was supported by the Key Project of Scientific and Technological Support Plan of Tianjin in 2020 (No.20YFZCSY00820).

## Conflict of Interest

The authors declare that the research was conducted in the absence of any commercial or financial relationships that could be construed as a potential conflict of interest.

## Publisher’s Note

All claims expressed in this article are solely those of the authors and do not necessarily represent those of their affiliated organizations, or those of the publisher, the editors and the reviewers. Any product that may be evaluated in this article, or claim that may be made by its manufacturer, is not guaranteed or endorsed by the publisher.
