# Supplementary material for: Impact of Metabolic Syndrome and Its Components on Clinical Severity and Long-Term Prognosis in Patients With Premature Myocardial Infarction
Source: Front Endocrinol (Lausanne). 2022 Jun 30;13:920470. doi: 10.3389/fendo.2022.920470 (PMC9279730; doi:10.3389/fendo.2022.920470)
Supplement: Supplementary file 1 [file DataSheet_1.docx]

**Supplementary Table**：Baseline data for major adverse cardiovascular events analysis

| **Variables** | All patients  （n=772） | Lost to follow-up  （n=63） | *P* | MACE at 42 months | | *P* |
| --- | --- | --- | --- | --- | --- | --- |
|  |  |  |  | Yes (n=102) | No (n=607) |  |
| Age (year) | 41(37-44) | 42（36-45） | 0.092 | 41（36-44） | 41（37-43） | 0.818 |
| Male ,n(%) | 740（95.9） | 60（95.2） | 0.814 | 95（93.1） | 585（96.4） | 0.127 |
| BMI(kg/m^2^) | 25.5(23.4-27.2) | 25.8（23.3-28.3） | 0.158 | 25.0（23.4-27.7） | 25.4（23.4-27.0） | 0.269 |
| **medical history** |  |  |  |  |  |  |
| History of Diabetes, n,(%) | 162（21.0）） | 11（17.5） | 0.507 | 29（28.4） | 122（20.1） | 0.057 |
| History of Hypertension, n,(%) | 366（47.4） | 32（50.8） | 0.605 | 48（47.1） | 286（47.1） | 0.991 |
| Previous angina pectoris, n,(%) | 172（22.3） | 9（14.3） | 0.139 | 26（25.5） | 137（22.6） | 0.517 |
| History of MI, n,(%) | 36（4.7） | 4（6.3） | 0.547 | 8(7.8) | 24（4.0） | 0.080 |
| Family history of CVD ,n,(%) | 117（15.2） | 7（11.1） | 0.385 | 15(14.7) | 95（15.7） | 0.807 |
| History of smoking ,n,(%) | 604（78.2） | 54（85.7） | 0.163 | 80（78.4） | 470（77.4） | 0.822 |
| History of drinking ,n,(%) | 322（41.7） | 31（49.2） | 0.247 | 42（41.2） | 249（41.0） | 0.977 |
| Renal insufficiency ,n,(%) | 10（1.3） | 0 | 0.363 | 1（1.0） | 9（1.5） | 0.691 |
| Previous PCI ,n,(%) | 23（3.0） | 3（4.8） | 0.433 | 6（5.9） | 14(2.3) | 0.044 |
| Previous CABG ,n,(%) | 3（0.4） | 0 | 0.620 | 2（2.0） | 1（0.2） | 0.010 |
| Cerebrovascular disease ,n,(%) | 24（3.1） | 1（1.6） | 0.496 | 7（6.9） | 16（2.6） | 0.026 |
| **Admission** |  |  |  |  |  |  |
| systolic blood pressure (mmHg) | 130.0(119.0-143.0) | 134.0（120.0-145.0） | 0.114 | 132（120-146） | 130.0（118.0-140.0） | 0.267 |
| **Type of MI** |  |  |  |  |  |  |
| STEMI, n,(%) | 558（72.3） | 48（76.2） | 0.504 | 79（77.5） | 431（71） | 0.180 |
| NSTEMI, n,(%) | 214（27.7） | 15（23.8） | 0.504 | 23（22.5） | 176（29.0） | 0.180 |
| **Laboratory** |  |  |  |  |  |  |
| LVEF＜40%, n,(%) | 44（6.0） | 4（6.6） | 0.861 | 15（15.6） | 25（4.3） | *P*＜0.001 |
| LVEF (%) | 53.0(47.0-58.0) | 51.0（45.5-57.0） | 0.224 | 50（43-57） | 53（48-58） | 0.003 |
| Fasting blood glucose (mmol/L) | 5.6(4.9-7.3) | 5.8（5.1-7.8） | 0.226 | 6.6（5.5-8.7） | 5.5（4.9-6.8） | *P*＜0.001 |
| CK(U/L) | 798.0(204.0-1992.0) | 975.0（385.0-1827.0） | 0.861 | 1570（626-3077） | 751（196.0-1847.5） | *P*＜0.001 |
| CK MB(U/L) | 70.0(24.3-170.0) | 77.0(29.0-130.0) | 0.857 | 121（46-227） | 65.5（24.0-165.0） | *P*＜0.001 |
| Hypersensitive C-reactive protein (mmol/L) | 5.5(2.5-14.9) | 5.0（1.8-11.6） | 0.537 | 5.7（3.4-18.7） | 5.4（2.2-14.5） | 0.020 |
| Total cholesterol (mmol/L) | 4.5（4.0-5.2） | 4.8（4.1-5.5） | 0.061 | 5.2±1.4 | 4.9±1.2 | 0.051 |
| Triglyceride (mmol/L) | 2.0(1.4-2.9) | 2.0（1.7-2.6） | 0.628 | 2.0（1.4-3.1） | 2.0（1.4-2.9） | 0.617 |
| HDL-C (mmol/L) | 0.9(0.8-1.1) | 0.9（0.8-1.1） | 0.599 | 0.9（0.8-1.0） | 0.9（0.8-1.1） | 0.588 |
| Hypersensitive troponin T(ug/L) | 1.4(0.4-4.0) | 1.5（0.6-5.1） | 0.681 | 3.2（1.0-6.1） | 1.2（0.4-3.9） | *P*＜0.001 |
| LDL-C (mmol/L) | 3.2(2.5-3.8) | 2.9（2.5-3.8） | 0.398 | 3.3（2.8-4.2） | 3.2（2.5-3.8） | 0.074 |
| **CAG and treatment** |  |  |  |  |  |  |
| CAG, n,(%) | 726（94.0） | 61（96.8） | 0.361 | 92（90.2） | 573（94.4） | 0.104 |
| Conservative treatment, n,(%) | 112（14.5） | 6（9.5） | 0.275 | 19（18.6） | 87（14.3） | 0.260 |
| Thrombolysis, n,(%) | 11（1.4） | 2（3.2） | 0.281 | 1（1.0） | 8（1.3） | 0.778 |
| CABG, n,(%) | 12（1.6） | 1（1.6） | 0.984 | 4（3.9） | 7（1.2） | 0.036 |
| PCI, n,(%) | 637（82.5） | 54（85.7） | 0.518 | 78（76.5） | 505（83.2） | 0.100 |
| Left main, n,(%) | 16（2.2） | 0 | 0.241 | 3（3.3）） | 13（2.3） | 0.564 |
| multi-vessel disease, n,(%) | 428（59.0） | 38（62.3） | 0.610 | 66（71.7） | 324（56.5） | 0.006 |
| Syntax score | 15.3（9.0-22.4） | 13.0（8.3-22.3） | 0.188 | 19（13-25.5） | 15.5（9.0-22.0） | 0.001 |
| Syntax≤22, n,(%) | 541（74.5） | 46（75.4） | 0.878 | 61（66.3） | 434（75.7） | 0.054 |
| Syntax（23-32）, n,(%) | 110（15.2） | 9（14.8） | 0.934 | 17（18.5） | 84（14.7） | 0.343 |
| Syntax≥33, n,(%) | 44（6.1） | 3（4.9） | 0.718 | 13（14.1） | 28（4.9） | 0.001 |
| Medication during follow-up |  |  |  |  |  |  |
| DAPT | 763（98.8） | 62（98.4） | 0.767 | 101（99.0） | 600（98.8） | 0.878 |
| Beta-blocker | 613（79.4） | 54（85.7） | 0.230 | 83（81.4） | 476（78.4） | 0.499 |
| ACEI/ARB | 515（66.7） | 48（76.2） | 0.123 | 70（68.6） | 397（65.4） | 0.525 |
| Statin | 763（98.8） | 63（100） | 0.389 | 101（99.0） | 599（98.7） | 0.778 |

Abbreviationsː MS group - patients with metabolic syndrome; non-MS group – patients without metabolic syndrome; MI – myocardial infarction; CVD - cardiovascular disease; PCI - percutaneous coronary intervention; CABG - coronary artery bypass graft; STEMI –ST-segment elevation myocardial infarction; NSTEMI –non-ST segment elevation myocardial infarction; CK-Creatine kinase; CK-MB – Creatine kinase isoenzyme; HDL-C - High density lipoprotein cholesterol; LDL-C- Low density lipoprotein cholesterol; CAG - coronary angiography


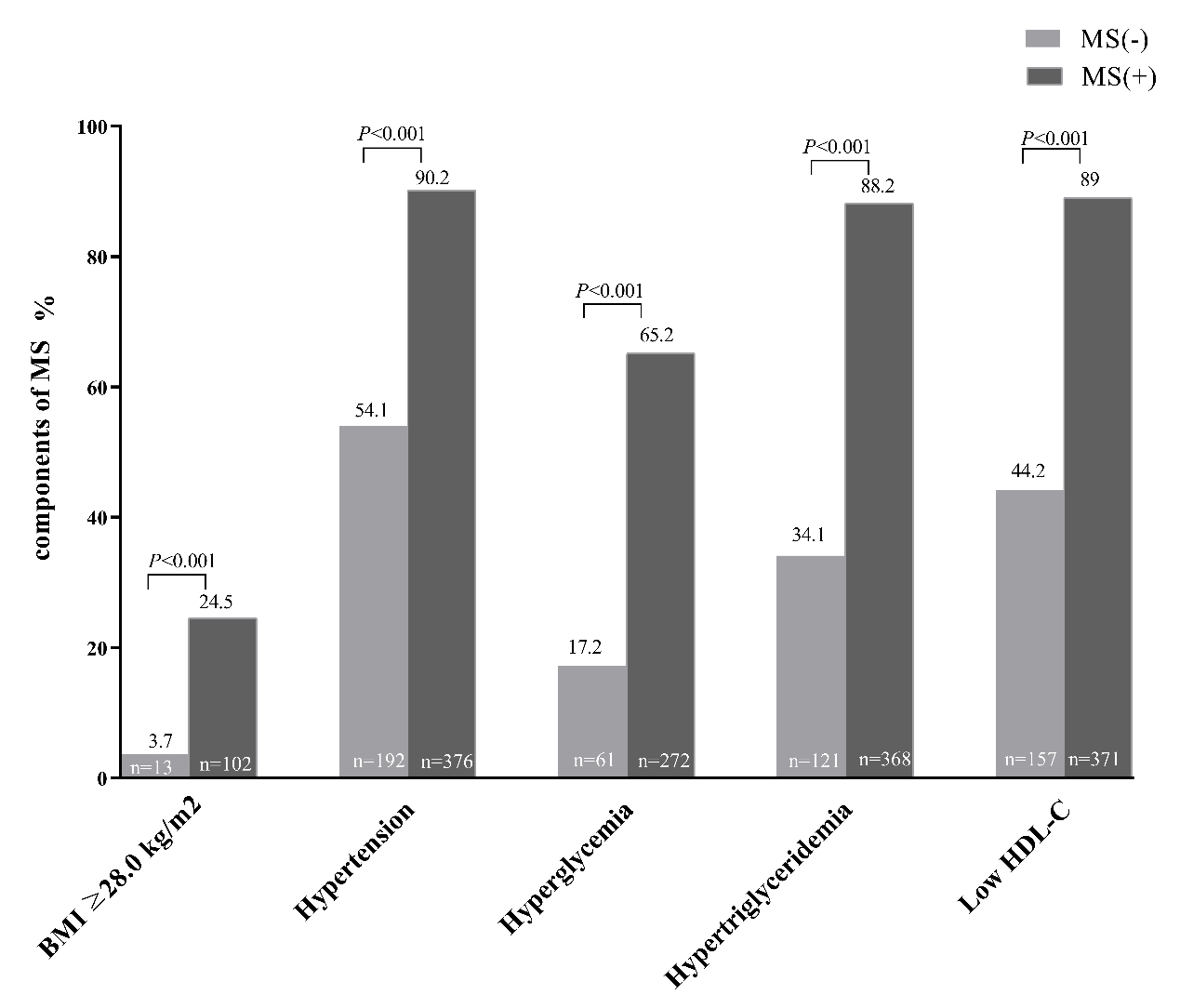


**Supplementary Figure . Comparison of five components of MetS between MetS and non-MetS groups.**
